# Astrocytes and Microglia Exhibit Cell-Specific Ca^2+^ Signaling Dynamics in the Murine Spinal Cord

**DOI:** 10.3389/fnmol.2022.840948

**Published:** 2022-03-30

**Authors:** Phillip Rieder, Davide Gobbo, Gebhard Stopper, Anna Welle, Elisa Damo, Frank Kirchhoff, Anja Scheller

**Affiliations:** ^1^Department of Molecular Physiology, Center for Integrative Physiology and Molecular Medicine (CIPMM), University of Saarland, Homburg, Germany; ^2^Department of Genetics and Epigenetics, University of Saarland, Saarbrücken, Germany; ^3^Institute of Pharmacology, Medical Faculty Heidelberg, Heidelberg University, Heidelberg, Germany

**Keywords:** spinal cord, astrocytes, microglia, Ca^2+^, laminectomy, slice preparation, *in vivo*, 2-photon laser-scanning microscopy

## Abstract

The spinal cord is the main pathway connecting brain and peripheral nervous system. Its functionality relies on the orchestrated activity of both neurons and glial cells. To date, most advancement in understanding the spinal cord inner mechanisms has been made either by *in vivo* exposure of its dorsal surface through laminectomy or by acute *ex vivo* slice preparation, likely affecting spinal cord physiology in virtue of the necessary extensive manipulation of the spinal cord tissue. This is especially true of cells immediately responding to alterations of the surrounding environment, such as microglia and astrocytes, reacting within seconds or minutes and for up to several days after the original insult. Ca^2+^ signaling is considered one of the most immediate, versatile, and yet elusive cellular responses of glia. Here, we induced the cell-specific expression of the genetically encoded Ca^2+^ indicator GCaMP3 to evaluate spontaneous intracellular Ca^2+^ signaling in astrocytes and microglia. Ca^2+^ signals were then characterized in acute *ex vivo* (both gray and white matter) as well as in chronic *in vivo* (white matter) preparations using MSparkles, a MATLAB-based software for automatic detection and analysis of fluorescence events. As a result, we were able to segregate distinct astroglial and microglial Ca^2+^ signaling patterns along with method-specific Ca^2+^ signaling alterations, which must be taken into consideration in the reliable evaluation of any result obtained in physiological as well as pathological conditions. Our study revealed a high degree of Ca^2+^ signaling diversity in glial cells of the murine spinal cord, thus adding to the current knowledge of the astonishing glial heterogeneity and cell-specific Ca^2+^ dynamics in non-neuronal networks.

## Introduction

The spinal cord is a highly sophisticated structure required for correct and rapid information transmission and processing (Bican et al., 2013; DeSai et al., 2021; Harrow-Mortelliti et al., 2021). In transverse sections, the spinal cord displays a distinct compartmentalization into white matter tracts containing neuronal fibers and a central gray matter region containing most of the neuronal cell bodies (Anderson et al., 2009). In both regions glial cells ensure alongside neurons reliable spinal network function and response upon physiological (such as somatosensory inputs) or pathological (such as neuropathic pain, inflammation, and spinal cord injury) stimuli. The attribution of glial cells to the correct functioning central nervous system has recently led to the comprehensive concept of an *active milieu* to point at the dynamic and reciprocal interactions between neuronal and glial compartments as well as extracellular space, extracellular matrix and vasculature that occur at any time within the nervous tissue (Semyanov and Verkhratsky, 2021). In particular, spinal cord injury induces robust alterations in both astroglial and microglial cellular phenotypes and activity around the lesion (Gaudet and Fonken, 2018; Hassanzadeh et al., 2021). Acute and sub-acute triggered, astroglial response fully develops in the range of days after the insult (Silver and Miller, 2004; Okada et al., 2006; Sofroniew and Vinters, 2010; Cregg et al., 2014; Fan et al., 2016; Liddelow et al., 2017; Li et al., 2019, 2020), whereas microglia can react in the scale of seconds or minutes (Prewitt et al., 1997; Dibaj et al., 2010; David and Kroner, 2011; Kopper and Gensel, 2018; Bellver-Landete et al., 2019; Kolos and Korzhevskii, 2020).

It is known since decades that glial cells undergo complex changes of internal Ca^2+^ concentration ([Ca^2+^]_*i*_), which represent a key read-out of glial activity and reactivity (Verkhratsky et al., 1998; Verkhratsky, 2006). In particular, astroglia exhibit highly dynamic intra- as well as intercellular Ca^2+^ signaling (Khakh and McCarthy, 2015; Caudal et al., 2020), both spontaneously (Araque et al., 1999; Parri et al., 2001; Nett et al., 2002; Haustein et al., 2014; Bindocci et al., 2017) as well as in response to extracellular inputs (Bazargani and Attwell, 2016; Panatier and Robitaille, 2016; Perea et al., 2016; Mariotti et al., 2018; Nagai et al., 2019; Kofuji and Araque, 2020). Importantly, neuropathological conditions (Bezzi et al., 2001; Rossi et al., 2005; Kuchibhotla et al., 2009; Carmignoto and Haydon, 2012; Hamby et al., 2012; Lee et al., 2013; Jiang et al., 2016; Mizuno et al., 2018; Shigetomi et al., 2019) as well as mechanical and biochemical insults (Shigetomi et al., 2019) perturb astroglial Ca^2+^ signaling, which in turn can mediate the cellular response and reactive phenotype. Microglia also display fast [Ca^2+^]_*i*_ changes (from a millisecond and up to minute range) due to the presence of Ca^2+^-permeable membrane ion channels (Möller, 2002; Kettenmann et al., 2011) as well as in response to a plethora of extracellular ligands (Ferrari et al., 1996; Nolte et al., 1996; Möller et al., 1997; Toescu et al., 1998; Biber et al., 1999; Visentin et al., 1999; Noda et al., 2000; Kuhn et al., 2004; Bianco et al., 2005; Light et al., 2006). Microglial Ca^2+^ signals correlate *in situ* with microglial reactive phenotype (Hoffmann et al., 2003; Färber and Kettenmann, 2006; Haynes et al., 2006; Ikeda et al., 2013; Heo et al., 2015; Michaelis et al., 2015; Korvers et al., 2016) and were recorded *in vivo* at low frequency, in response to damage-induced ATP release (Eichhoff et al., 2011; Pozner et al., 2015; Brawek et al., 2017). Notably, hypoactive shifts in neuronal activity (Brawek et al., 2014; Umpierre et al., 2020) as well as neuronal hyperactivity during kainate induced-status epilepticus and after chemogenetic artificial activation (Umpierre et al., 2020) increase the frequency of microglial Ca^2+^ signals *in vivo*. Nevertheless, as for astroglia, the microglial reactive phenotype and subsequent inflammatory response involve [Ca^2+^]_*i*_ variations, which may add to different extents on the microglial contribution to several pathophysiological conditions (Glass et al., 2010; Perry et al., 2010; Kettenmann et al., 2011; Brawek and Garaschuk, 2013; Ransohoff and El Khoury, 2015).

To date, the detailed characterization of glial Ca^2+^ signaling in the spinal cord has been facing up to its limited accessibility and the extensive manipulation required to either obtain acute slice preparations or perform acute and chronic *in vivo* imaging (Cupido et al., 2014; Cartarozzi et al., 2018; Nelson et al., 2019). In comparison to spinal cord neuronal Ca^2+^ signaling (Johannssen and Helmchen, 2010; Nishida et al., 2014; Sekiguchi et al., 2016), little is known about astroglial Ca^2+^ activity (Cirillo et al., 2012; Sekiguchi et al., 2016) and none, to our knowledge, about spinal microglial Ca^2+^ activity. It is therefore unknown if under physiological conditions these glial cell types display similar Ca^2+^ changes to other CNS regions or whether they exhibit distinct specifications. In addition, it needs to be elucidated to which extent their activity is affected by the experimental procedure required to access them, i.e., acute slice preparation (*ex vivo*) and chronic window implantation for *in vivo* imaging. Here, we provide a comprehensive analysis of Ca^2+^ signals in astroglia and microglia of the murine spinal cord using transgenic mice with cell-type specific expression of a genetically encoded Ca^2+^ indicator (GCaMP3), thus adding on the long-lasting and still ongoing research on the heterogeneity of glial Ca^2+^ signaling.

## Materials and Methods

### Animals

Mice were maintained in the animal facility of the Center for Integrative Physiology and Molecular Medicine (CIPMM, University of Saarland, Homburg). Humidity and temperature were maintained at 45–65% and 20–24°C and the facility was kept under a 12 h light-dark cycle. All mice received food *ad libitum* (standard autoclaved rodent diet, Ssniff Spezialdiäten, Soest, Germany) and autoclaved tap water. Transgenic hGFAP-Cre^ERT2^ mice [Tg(GFAP-cre/ERT2)1Fki, MGI:4418665] (Hirrlinger et al., 2006) and knock-in CX_3_CR_1_-Cre^ERT2^ mice [Cx3cr1^tm2.1(cre/ERT2)Jung^, MGI: 5467985] (Yona et al., 2013) were crossbred to mice with Rosa26 reporter mice [Gt(ROSA)26Sor^tm1(CAG–GCaMP3)Dbe^, MGI: 5659933] (Paukert et al., 2014). To induce GCaMP3 expression, tamoxifen was administered intraperitoneally for three consecutive days (once per day, 100 mg/kg body weight) (Jahn et al., 2018) at 10 weeks of age. Spinal cord laminectomy, acute slice preparation, 2P-LSM and IHC were performed at 12–13 weeks of age.

### Laminectomy and Spinal Window Implantation

All surgical sections were realized in animals under inhalational anesthesia (1.5–2% isoflurane, 66% O_2_ and 33% N_2_O) and the animal’s eyes were covered by Bepanthen (Bayer, Leverkusen, Germany). Surgeries were adapted and modified from Fenrich et al. (2012) to get access to T12-L2 vertebrae and by laminectomy approach, L4-S1 spinal segments could be exposed. For chronic observations, a modified coverslip was fit on the spinal cord and animals were postoperatively injected subcutaneously with analgesic and antiphlogistic agents for two consecutive days (Cupido et al., 2014).

### Acute Spinal Cord Slice Preparation

After cervical dislocation, spinal T13-L1 segments were dissected and further processed in ice-cold artificial cerebrospinal fluid [aCSF; in mM, 125 NaCl, 2.5 KCl, 2 CaCl_2_, 1 MgCl_2_, 1.25 NaH_2_PO_4_, 25 NaHCO_3_, and 25 D-glucose, 330 mOsm/l, pH 7.4; adapted from Hirrlinger et al. (2005)] and purged by carbogen. Afterward, longitudinal sections were cut by a vibratome (VT1200 S) (Leica, Nußloch, Germany) with 250 μm thickness, primarily maintained at 37°C for 30 min and subsequently stored at room temperature for further 30 min and during 2P-LSM.

### Two-Photon Laser-Scanning Microscopy

To perform *in vivo* and *ex vivo* recordings, a custom-made two-photon laser-scanning microscope (2P-LSM), equipped with a mode-locked Ti:sapphire femto second pulsed laser, Vision II (Coherent, St. Clara, United States) (Dibaj et al., 2010), in combination with ScanImage software (Pologruto et al., 2003) was used. For transgenic GCaMP3 excitation, the laser wavelength was set to 890 nm and the power was adjusted from 8 to 60 mW, depending on the imaging depth in the tissue. 2P-LSM was performed on the white matter of the dorsal funiculus for *in vivo* and *ex vivo* preparations as well as on the dorsal horn gray matter for *ex vivo* preparations up to a 100–150 μm depth (laminae IV and V) by using a long-distance W Plan-Apochromat 20 × 1.0 NA DIC objective (Zeiss, Oberkochen, Germany). Areas of white and gray matter were recorded as uniformly spaced planes of field of views with 256 × 256 pixel per image, 1.4 μs pixel dwell time and GCaMP3 emission was acquired using a 500/24 nm band pass filter, detected by a photomultiplier tube H10770PB-40 (Hamamatsu Photonics, Hamamatsu, Japan). During 2P-LSM *ex vivo* spinal cord slices were continuously perfused with carbogenated aCSF.

### Automated ROA-Based Detection and Analysis of Ca^2+^ Events

Ca^2+^-event analysis was performed using a custom-made analysis software, developed in MATLAB (MSparkles, unpublished). Fluorescence fluctuations at basal Ca^2+^ concentrations (F_0_) were computed along the temporal axes of each individual pixel, by fitting a polynomial of user-defined degree in a least-squares sense. Prior to polynomial fitting, potential Ca^2+^ signals were removed for the purpose of F_0_ estimation. The range projection of ΔF/F_0_ was then used to identify local fluorescence maxima, serving as seed points for simultaneous, correlation-based region growing. Therefore, the correlation of a candidate pixel’s fluorescence profile with the fluorescence profile of its corresponding seed point was computed, using Pearsons’s linear correlation coefficient. A user-definable correlation threshold was used to stop the region growing process as soon as the temporal evolution of a candidate pixel deviated from its respective seed point (minimum ROA area, 5 μm^2^; temporal correlation threshold, 0.2). Pixels belonging to two adjacent regions were marked as boundary pixels. Prior to F_0_ estimation, image stacks were denoised using the PURE-LET algorithm (Luisier et al., 2011) as well as a temporal median filter to correct small motion artifacts and simultaneously retain sharp transient edges. Based on the pre-processed data (F), Ca^2+^ event detection and analysis were performed on the normalized dataset (ΔF/F_0_) (Table 1). MSparkles automatically computed Ca^2+^ signal parameters, such as peak amplitude, duration, Ca^2+^ signal start and end time, ROA area and per-ROA signal frequency. The fluorescence profiles of each ROA were obtained by computing the mean fluorescence among the ROA pixels per recorded time point. ROA areas were obtained by reading the pixel sizes from the image metadata and multiplying them with the individual number of pixels per ROA. Signal durations were computed at full-width at half-maximum (FWHM) of a signal’s peak amplitude. Start and end times of a signal were computed as the intersection points of the FWHM with the transient curve. Per ROA signal frequency was computed only if more than one signal was detected within a ROA as the mean signal frequency.

**TABLE 1:** Description of parameters used for Ca^2+^ characterization.

Feature	Description	Unit
Amplitude	Local maximum peak within a ROA	ΔF/F_0_
Area	Area covered by a ROA in domain units	μm^2^
Decay time	Time interval between 90% of the peak value and signal end	s
Duration	Full Width at Half Maximum (FWHM) of the signal curve	s
Frequency	Signal frequency within a chosen ROA based on the mean value of all peak-to-peak times divided by the number of signals associated to that ROA	min^–1^
Integrated fluorescence	Area under the signal curve in correspondence of the signal duration	ΔF/F_0_
Rise time	Time interval between signal start and 90% of the peak value	s
ROA density	Number of ROAs detected within a Field of View (FOV) divided by the FOV area	10^–3^/μm^2^
Signal density	Number of signals detected within a FOV divided by the FOV area	10^–3^/μm^2^
Signal start/end	Intersections of the signal curve with the horizontal line corresponding to 50% of the signal peak amplitude	
Coincidence index	Number of simultaneously active ROAs within a FOV normalized to the total number of ROAs	*c.i.*

### Immunohistochemistry

Anesthetized animals were transcardially perfused with phosphate-buffered saline (PBS) and tissue was fixed by 4% formaldehyde perfusion. After 24 h post fixation in 4% formaldehyde, T13-L1 spinal cord segments were dissected and detached from meninges. The spinal cord tissue was maintained in PBS and cut in transversal or longitudinal sections (40 μm) by vibratome (VT1000 S) (Leica, Nußloch, Germany). Free floating slices were processed for immunohistochemistry (IHC) as described before (Huang et al., 2020). Briefly, incubation in blocking solution (Triton X-100, horse serum and PBS) at RT was followed by primary antibody solution incubation overnight at 4°C for detection of the following glial markers: monoclonal mouse: anti-GFAP (1:500, Novacostra, NCL-GFAP-GA5), anti-GFP (1:500, Abcam, ab1218), polyclonal goat: anti-GFAP (1:1,000, Abcam, ab53554), anti-Iba1 (1:1,000, Abcam, ab5076), polyclonal rabbit: anti-GFP (1:1,000, Clontech, 632593), anti-Iba1 (1:1,000, Wako, 019-19741). Detection of the secondary antibodies (donkey anti-mouse, goat and rabbit secondary antibodies conjugated with Alexa488, Alexa555, Alexa633 and Alexa647; 1:2.000 in PBS; Invitrogen, Grand Island, NY, United States) was executed with the fully automated epifluorescence slide scanner microscope AxioScan.Z1 using the Colibri 7 LED system and appropriate filters (Zeiss, Oberkochen, Germany). Image stacks (5 μm, variance projection) were recorded and analyzed with ZEN blue (Zeiss, Oberkochen, Germany).

### Software

For 2P-LSM acquisition, the open-source MATLAB-based software application ScanImage^®^ (Vidrio Technologies, Ashburn, VA, United States) (Pologruto et al., 2003) was used. The custom-made MATLAB-based software MSparkles, GraphPad Prism 8 and Microsoft Office Excel 2016 were used for data analysis. Immunohistochemical data were visualized and modified using the ZEN blue imaging software (Zeiss, Oberkochen, Germany) and the ImageJ collection Fiji. For figure layout, the Adobe Creative Suite 2021 was used (Adobe InDesign^®^, Adobe Illustrator^®^, Adobe Photoshop^®^).

### Unsupervised Clustering Analysis

Clustering analysis was performed using MSparkles output as medians of all signals at Field of View (FOV) level. The data was imported into R Studio R Studio Team (2020). RStudio: Integrated Development for R. RStudio, PBC, Boston, MA URL^1^. Heatmaps of scaled values were generated using the R package *pheatmap()^2^*.

### Statistics

Unless otherwise stated, data are represented as mean ± SEM of single FOVs. Single datasets were analyzed using a Shapiro–Wilk normality test and represented as FOV medians. Data were compared using an ordinary one-way ANOVA with Bonferroni’s multiple comparisons test. Non-linear fitting of the data was performed using a Least-Squares fitting with no weighting method and compared using the extra-sum-of-squares F test. F ratios and relative *p-values* of single curve comparisons are schematically represented as a polygonal diagram and gray-scaled color-coded. For paired comparisons, a Wilcoxon matched pairs signed rank test was used. For statistical analysis, following *p-values* were used: *p < 0.05; ^**^p < 0.01; ^***^p < 0.001, ^****^p < 0.0001.

### Ethics Statement

All animal experiments were performed at the University of Saarland, Center for Integrative Physiology and Molecular Medicine (CIPMM), in strict accordance with the recommendations to European and German guidelines for the welfare of experimental animals and approved by the “Landesamt für Gesundheit und Verbraucherschutz” of the state of Saarland (animal license number 34/2016, 36/2016, 03/2021 and 08/2021). For 2P-LSM *N* ≥ 4 animals were used for each *ex vivo* or *in vivo* experiment (total 20 animals), for immunohistochemical analysis 24 animals were used in total.

## Results

### Tamoxifen-Induced GCaMP3 Expression in Adult Spinal Cord Astro- and Microglia and Acquisition of Ca^2+^ Signals

**FIGURE 1:**
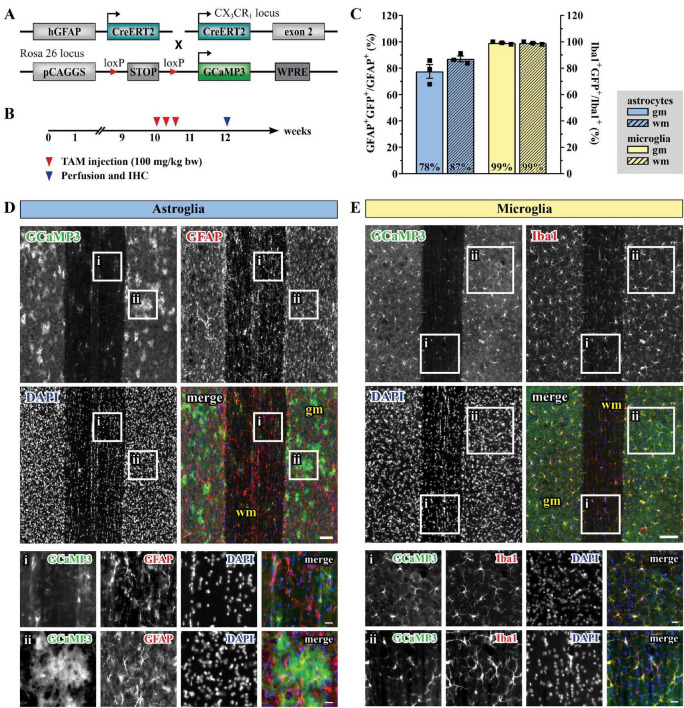
Tamoxifen- induced GCaMP3 expression in astrocytes and microglia. **(A)** Scheme of transgenic constructs carrying CreERT2 and GCaMP3. **(B)** Experimental design for the GCaMP3 induction in 10-weeks old mice injected with TAM (tamoxifen). **(C)** Recombination efficiency for hGFAP^GCaMP3^ (blue, left Y-axis) and CX_3_CR_1_^GCaMP3^ animals (yellow, right Y-axis) in gray (*gm*; solid) and white matter (*wm*, dashed), respectively. **(D)** Evaluation of longitudinal spinal cord slices for cell-specific GCaMP3 (green) expression in astroglia (GFAP, red) and **(E)** microglia (Iba1, red) with nucleic staining (DAPI, blue). Neither Iba1^+^GCaMP3^+^ nor GFAP^+^ GCaMP3^+^ cells were detected in the hGFAPCre^ERT2^ or CX_3_CR_1_-Cre^ERT2^ animals, respectively. **(Di,Ei)** *wm*, magnified view; **(Dii,Eii)** *gm*, magnified view. Scale bar, 100 μm (overviews) and 20 μm (magnified views).

In order to record astroglial and microglial Ca^2+^ variations, we took advantage of the inducible DNA recombinase CreERT2 to achieve time-controlled and cell type-specific expression of the genetically-encoded Ca^2+^indicator GCaMP3 (Paukert et al., 2014) in astroglia under the control of the human glial fibrillary acidic protein promoter (GFAP) and in C-X3-C motif chemokine receptor 1 (CX_3_CR_1_)-expressing microglia (Figure 1A; Hirrlinger et al., 2006; Yona et al., 2013). 10 weeks-old mice with C57BL6/N background were treated with tamoxifen (100 mg/kg body weight, i.p., three times, once per day) to induce the reporter expression in astroglia (for simplicity we will refer to these animals as hGFAP^GCaMP3^ mice) and microglia (CX_3_CR_1_^GCaMP3^ mice, Figure 1B). The cellular specificity of the recombination in both gray (*gm*) and white matter (*wm*) of the lumbar spinal cord was confirmed by immunohistochemistry and colocalization with GFAP (Figures 1C,D) and Iba1 (Figures 1C,E) in longitudinal spinal cord slices. Astroglia displayed the typical protoplasmic and fibrous morphology in *gm* and *wm*, with high recombination efficiencies (78% in *gm* and 87% in *wm* for GFAP^+^ cells). On the other hand, microglial population appeared morphologically homogeneous throughout the tissue with 99% recombination efficiency in *gm* and *wm* for Iba1^+^ cells. To visualize Ca^2+^ changes, 12 weeks-old animals were sacrificed for acute slice preparation and *ex vivo* two-photon laser-scanning microscopy (2P-LSM). Alternatively, they underwent a laminectomy surgery (T13 and L1 vertebrae) in order to expose the spinal cord segments L5 and L6 for chronic *in vivo* 2P-LSM one (*d1*), two (*d2*) and 7 days (*d7*) after surgery (Supplementary Figures 1A,B). The absence of an excessive and abnormal cellular reaction after spinal cord surgery was monitored by immunohistochemistry of GFAP and Iba1 in both hGFAP^GCaMP3^ and CX_3_CR_1_^GCaMP3^ mice at each time point of investigation (Supplementary Figure 1C).

### Astroglial Ca^2+^ Signals Appear at Higher Densities Than Microglia in Acute Slice Preparations

Acute longitudinal slice preparations enabled access to both *gm* and *wm* of the spinal cord. In this work, we recorded glial Ca^2+^ signals from the dorsal *wm* and the dorsal horn *gm* and compared them with *in vivo* data collected in the dorsal spinal cord. Astro- and microglial Ca^2+^ data were processed and analyzed using the MATLAB-based analysis software MSparkles (Table 1 and see section “Materials and Methods” for details), which performed automatic and unbiased detection of regions of activity (ROAs) based on the range projection of ΔF/F_0_ and a temporal correlation based region detection algorithm. This approach enabled the detection of stationary ROAs associated with time-dependent fluorescence fluctuations, in contrast to other approaches based on dynamic events with different occurrence, extension and location over time (Cornell-Bell et al., 1990; Jung et al., 1998; Wu et al., 2014; Semyanov et al., 2020). For simplification and a comparison at a glance we collected all numeric values in Table 2. Throughout this work we will focus on the parameters that enable a clearer segregation of glial Ca^2+^ signals, namely signal and ROA density, ROA area, signal frequency and coincidence (in bold in Table 2).

**TABLE 2:** Numeric values of Ca^2+^ data in terms of morphology, spatial and temporal distribution (*mean* ± *SEM*).

	*ex vivo*		*in vivo*		
Astrocytes					
	gm	wm	wm		
			d1	d2	d7
Amplitude (ΔF/F_0_)	0.14 ± 0.01	0.13 ± 0.01	0.15 ± 0.02	0.14 ± 0.01	0.16 ± 0.01
Coincidence index (c.i.)	**0.07 ± 0.01**	**0.05 ± 0.01**	**0.03 ± 0.01**	**0.04 ± 0.01**	**0.03 ± 0.01**
Decay Time (s)	3.46 ± 0.55	2.32 ± 0.33	2.91 ± 0.49	3.26 ± 0.39	3.49 ± 0.56
Duration (s)	9.06 ± 1.40	5.93 ± 0.76	7.54 ± 1.12	9.21 ± 1.47	7.88 ± 1.14
Integrated fluorescence (ΔF/F_0_)	1.60 ± 0.20	1.03 ± 0.14	1.47 ± 0.26	1.62 ± 0.21	1.70 ± 0.36
Relative ROA frequency (%)	**52.80 ± 6.11**	**44.79 ± 2.04**	**68.24 ± 6.13**	**67.07 ± 4.86**	**61.29 ± 8.95**
Rise Time (s)	2.44 ± 0.32	1.75 ± 0.14	2.40 ± 0.50	3.41 ± 1.04	2.07 ± 0.49
ROA area (μm^2^)	**36.70 ± 5.02**	**18.61 ± 2.96**	**134.8 ± 33.16**	**102.4 ± 12.26**	**178.5 ± 49.87**
ROA density (10^–3^/μm^2^)	**3.18 ± 0.35**	**3.18 ± 0.33**	**1.83 ± 0.50**	**1.25 ± 0.21**	**0.89 ± 0.30**
Signal density (10^–3^/μm^2^)	**6.60 ± 1.42**	**7.68 ± 1.28**	**3.48 ± 1.17**	**2.47 ± 0.52**	**2.39 ± 1.07**
Signal frequency (min^–1^)	0.93 ± 0.06	1.11 ± 0.06	1.05 ± 0.13	1.40 ± 0.19	1.27 ± 0.18

	***ex vivo***		***in vivo***		
**Microglia**					
	**gm**	**wm**	**wm**		
			**d1**	**d2**	**d7**

Amplitude (ΔF/F_0_)	0.14 ± 0.01	0.14 ± 0.01	0.14 ± 0.00	0.13 ± 0.00	0.17 ± 0.02
Coincidence index (c.i.)	**0.04 ± 0.01**	**0.08 ± 0.01**	**0.10 ± 0.01**	**0.09 ± 0.01**	**0.10 ± 0.02**
Decay Time (s)	1.95 ± 0.19	2.33 ± 0.19	3.84 ± 0.59	2.75 ± 0.34	2.76 ± 0.41
Duration (s)	4.97 ± 0.38	6.18 ± 0.46	10.20 ± 1.39	7.29 ± 0.72	7.33 ± 0.93
Integrated fluorescence (ΔF/F_0_)	1.37 ± 0.19	1.41 ± 0.16	1.57 ± 0.18	1.16 ± 0.12	1.68 ± 0.38
Relative ROA frequency (%)	**56.40 ± 4.10**	**47.57 ± 3.84**	**36.82 ± 4.55**	**41.83 ± 5.17**	**29.10 ± 5.81**
Rise Time (s)	1.63 ± 0.16	2.04 ± 0.13	3.55 ± 0.57	2.32 ± 0.22	1.67 ± 0.14
ROA area (μm^2^)	**15.16 ± 2.99**	**12.37 ± 2.04**	**47.52 ± 4.02**	**52.17 ± 7.97**	**122.0 ± 13.91**
ROA density (10^–3^/μm^2^)	**1.23 ± 0.30**	**1.31 ± 0.20**	**3.00 ± 0.29**	**2.78 ± 0.26**	**2.79 ± 0.27**
Signal density (10^–3^/μm^2^)	**2.15 ± 0.57**	**2.81 ± 0.49**	**9.08 ± 1.56**	**8.33 ± 1.54**	**8.70 ± 1.34**
Signal frequency (min^–1^)	1.09 ± 0.14	1.05 ± 0.04	0.95 ± 0.08	0.97 ± 0.09	1.20 ± 0.08

*Highlighted values refer to parameters enabling clear data segregation.*

**FIGURE 2:**
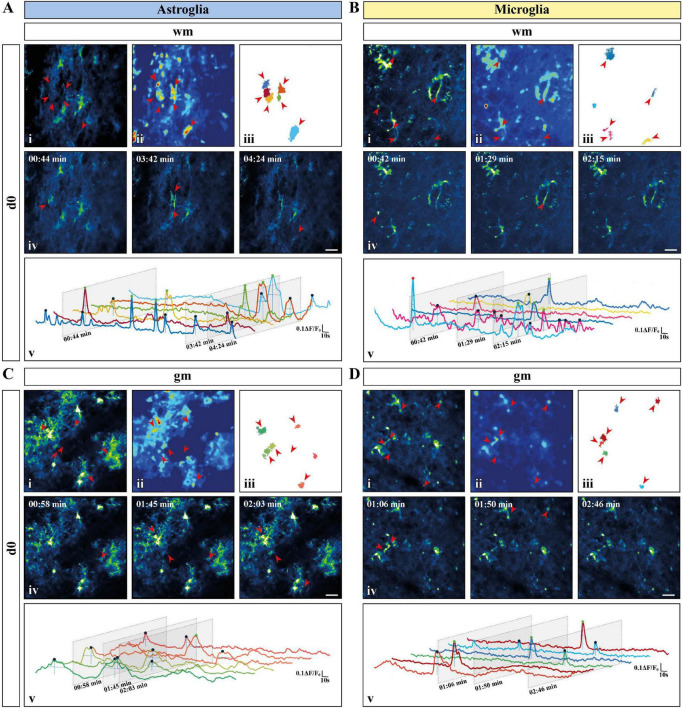
Activity-based Ca^2+^ signaling analysis for astroglia and microglia from acute preparations. **(A–D)** Representative Ca^2+^ signaling analysis for astroglia [**(A)** white matter (*wm*); **(C)** gray matter (*gm*)] and microglia [**(B)** *wm*; **(D)** *gm*] in acute slice preparations using the custom-made MATLAB-based software MSparkles. Maximum-intensity projection of the GCaMP3 signal for representative Fields of View [FOV; scale bar, 20 μm; (i)] over the entire recording time (up to 5 min), absolute intensity projection (ii) and selected regions of activity [ROAs; (iii)] automatically detected using variations in absolute intensity. Red arrows indicate the locations of selected ROAs. Representative time frames from the selected recordings [scale bar, 20 μm; (iv)] with red arrows indicating location of the selected ROAs if active in the displayed time frame. Normalized relative fluorescence intensity traces over time (ΔF/F_0_) for the selected ROAs (v) with trace colors matching colors of the selected ROAs. Oblique sections indicate time points chosen for display. Automatically detected signals were pinpointed and color coded based on signal strength (μ + σ ≤ ΔF/F_0_ ≤ μ + 2σ, blue; μ + 2σ < ΔF/F_0_ ≤ μ + 3σ, green; ΔF/F_0_ > μ + 3σ, red) calculated on the mean value (μ) and the corresponding standard deviation (σ) over all ROAs.

**FIGURE 3:**
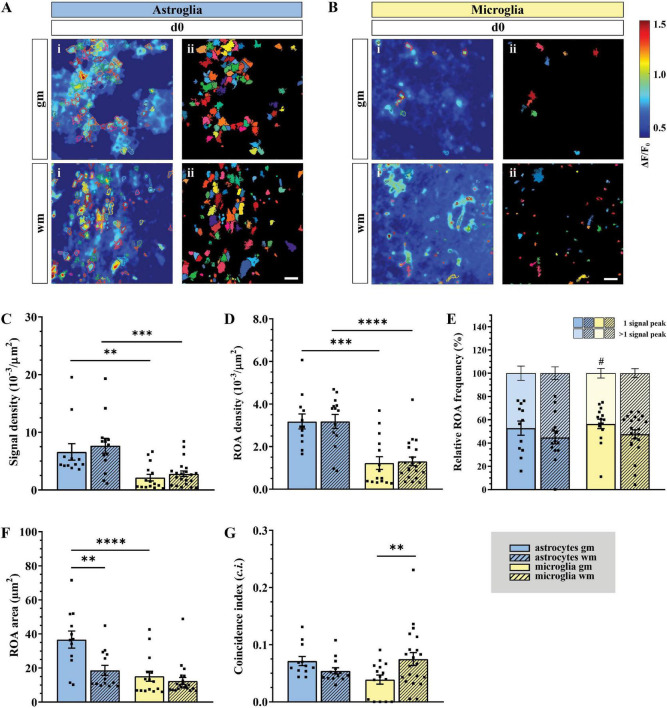
Higher astroglial signal and ROA density in gray and white matter of acute slice preparations (*ex vivo*). **(A,B)** Automatic analysis of detected regions of activity (ROAs) based on absolute fluorescence projection along the temporal axis for astroglia [**(A)** blue] and microglia [**(B)** yellow] in *gm* and *wm* of acute slice preparations (*d0*) with absolute fluorescence projections (i) overlapped with the edges of the detected ROAs [depicted as solid areas in panel (ii)]. **(C)** Signal and **(D)** ROA density in *gm* (solid) and *wm* (dashed). **(E)** Relative frequency of ROAs active once (lower bars) or more than once (upper bars) over the recording time. **(F)** ROA area and **(G)** mean relative number of simultaneously active ROAs over the total recording time calculated as coincidence index (*c.i.*). Data represented as mean ± SEM of single FOVs. Single datasets were analyzed using a Shapiro–Wilk normality test and represented as FOV medians. **(C–G)** Data were compared using an Ordinary one-way ANOVA with Bonferroni’s multiple comparisons test. For each group in panel **(E)**, the relative numbers of ROAs presenting one or more signal peaks were analyzed using a Wilcoxon matched pairs signed rank test. *N* (animals) = 4 (astroglia), 5 (microglia, *gm*), 6 (microglia, *wm*). *n* (FOVs) = 12 (astroglia, *gm*), 14 (astroglia, *wm*), 15 (microglia, *gm*), 22 (microglia, *wm*)., ^#^*p* < 0.05; ^**^p < 0.01; ^***^p < 0.001; ^****^p < 0.0001.

*Ex vivo* astroglial Ca^2+^ imaging confirmed the morphological differences between *gm* and *wm* astrocytes observed in fixed tissue preparations and the expression of GCaMP3 in the entire cellular cytoplasm (Figures 2A,C and Supplementary Videos 1, 2). Although similar between *wm* and *gm*, microglia exhibited morphological changes typical of their reactive phenotype with shorter and thicker processes and in some cases even an amoeboid cell body (Figures 2B,D and Supplementary Videos 3, 4). Notably, the majority of microglia shared this phenotype irrespective of the imaging depth. Both *gm* and *wm* astrocytes displayed highly dynamic Ca^2+^ oscillations mainly restricted to the gliapil but occasionally involving the somatic compartment, whereas microglia were mostly silent or displayed changes at a lower frequency and were often restricted to single branches. In line with this, the automatic ROA detection analysis revealed different signal density (10^–3^/μm^2^) between astroglia and microglia in both *gm* (*p* < 0.01) and *wm* (*p* < 0.001) with a ∼3-fold increase in signal density in astroglia compared to microglia (Figures 3A–C). No difference was detected within each cell-type between *gm* and *wm*. In parallel to that, both astroglia and microglia did not display any difference in ROA density (10^–3^/μm^2^) between *gm* and *wm*, whereas in both regions astroglia showed a ∼2.5-fold increase in ROA density (*gm*: *p* < 0.001; *wm*: *p* < 0.0001; Figure 3D). A closer look at the signal distribution among the detected ROAs revealed that around half of the ROAs were active only once during the recording time, irrespectively of the total number of active ROAs (Figure 3E). Also, the signal frequency for ROAs associated with more than one signal did not differ between regions and cell types and ranged between ∼0.50 and ∼1.50 min^–1^ for astroglia and ∼0.6 and ∼2.5 min^–1^ for microglia (Table 2).

In terms of the signal most obvious kinetic properties, namely the amplitude at their maximum peak (ΔF/F_0_) and the signal duration, defined as full-width at half maximum (FWHM), most astroglial and microglial signals displayed amplitudes ranging from ∼110 to ∼120% of the baseline fluorescence level with no difference between cell-types or regions. Astroglial Ca^2+^ signals lasted longer in *gm* than in *wm* (*p* < 0.05) as well as microglial *gm* signals (*p* < 0.01, Table 2). Additionally, we evaluated the distribution of the signal amplitudes and durations by sorting the signals according to their amplitude (Supplementary Figure 2A) or duration (Supplementary Figure 2B) and plotting their relative frequency. Next, we fitted the data with a lognormal distribution using a Least-Squares fitting and compared them using the extra-sum-of-squares F test. The relative frequency curve of the amplitudes of the Ca^2+^ changes recorded *ex vivo* was similar among different regions and between astroglia and microglia. With respect to the signal duration distribution, the oscillations of Ca^2+^ signals displayed higher variations between cell-types than between *gm* and *wm* (*p* < 0.0001). We also provide further analysis of the signal morphology in a two-dimensional space, namely the signal profile along the time axis, by evaluating the signal integrated fluorescence (ΔF/F_0_) as well as the rise and decay time (s) (Table 2). In line with the signal duration, astroglial *gm* changes displayed a higher integrated fluorescence as well as longer rise and decay times compared to astroglial signals in the *wm*, whereas microglial integrated fluorescence did not differ between the two regions.

In terms of spatial distribution of the Ca^2+^ elevations, microglial ROAs had the same area (μm^2^) in the dorsal *gm* and *wm*, whereas astroglial ROAs displayed a ∼2-fold increase in their extension in the *gm* (*p* < 0.01; Figure 3F). In line with this, the comparison of the relative frequency distributions of the ROA areas (Supplementary Figure 2C) confirmed that active astroglial ROAs in the *gm* were larger than in the *wm* (*p* < 0.0001) and microglial ROAs in the same region (*p* < 0.0001). Notably, microglia in the *gm* had a higher relative number of smaller ROAs compared to the *wm* (*p* < 0.0001). Finally, we analyzed the signals based on their coincident appearance and calculated the relative number of ROAs active at a given time point (coincidence index, *c.i.*, Figure 3G). We found that astroglial Ca^2+^ changes were similarly active between *gm* and *wm*, whereas microglial changes were less coincident in *gm* (*p* < 0.05).

### *In vivo* Microglial Ca^2+^ Changes Are Characterized by a Higher Density and Coincidence but Smaller Areas

**FIGURE 4:**
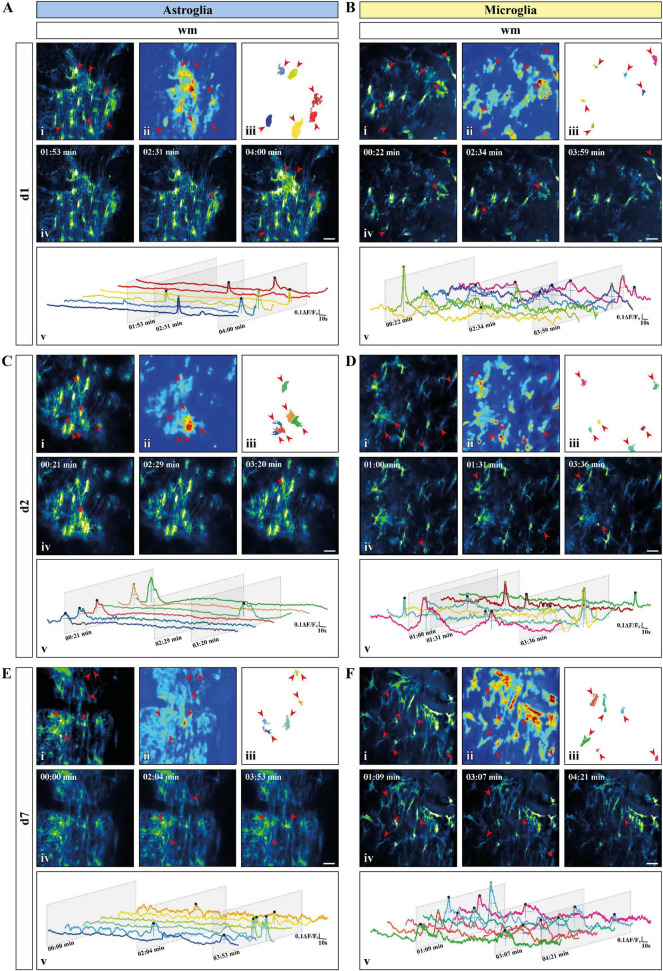
Activity-based *in vivo* Ca^2+^ signaling analysis for astro- and microglia after chronic window implantation. **(A–F)** Representative Ca^2+^ signaling analysis for **(A,C,E)** astroglia and **(B,D,F)** microglia monitored *in vivo* [**(A,B)** day one (*d1*); **(C,D)** day two (*d2*); **(E,F)** day 7 (*d7*)] using the custom-made MATLAB-based software MSparkles. Maximum-intensity projection of GCaMP3 signals for representative FOV [scale bar, 20 μm; (i)] over the entire recording time (up to 5 min), absolute intensity projection (ii) and selected regions of activity [ROAs; (iii)] automatically detected using variations in the absolute intensity. Red arrows indicate location of the selected ROAs with representative time frames from the selected recordings [scale bar, 20 μm; (iv)]. Red arrows indicate the location of the selected ROAs if active in the displayed time frame. Normalized relative fluorescence intensity traces over time (ΔF/F_0_) for the selected ROAs (v) with trace colors matching the colors of the selected ROAs and oblique sections indicating the time points chosen for display. Automatically detected signals were pinpointed and color coded based on signal strength (μ + σ ≤ ΔF/F_0_ ≤ μ + 2σ, blue; μ + 2σ < ΔF/F_0_ ≤ μ + 3σ, green; ΔF/F_0_ > μ + 3σ, red) calculated on the mean value (μ) and the corresponding standard deviation (σ) over all ROAs.

**FIGURE 5:**
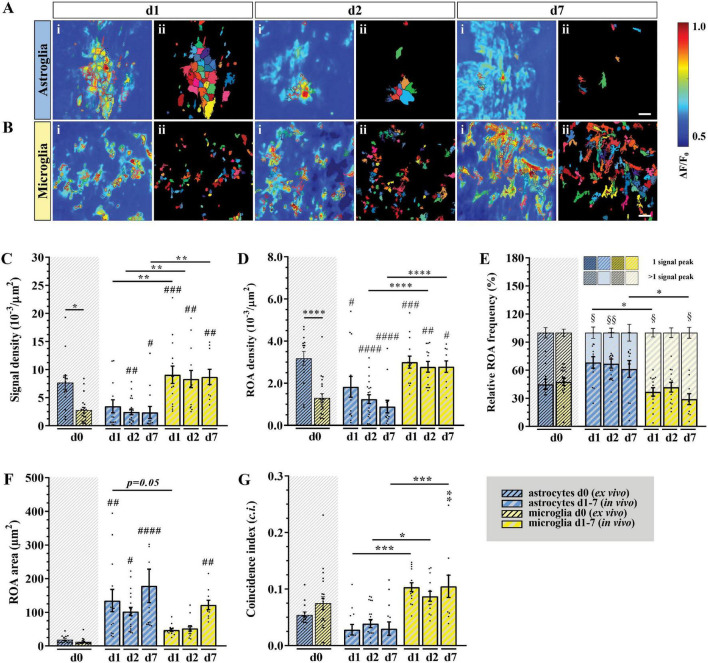
*In vivo* microglial Ca^2+^ signals showed higher signal and ROA densities as well as smaller areas and a higher temporal coincidence. **(A,B)** Automatic detected ROAs based on absolute fluorescence projections along the temporal axis for astroglia (blue) and microglia (yellow) *in vivo* (*d1*, *d2* and *d7*, *wm*). Absolute fluorescence projections (i) were overlapped with the edges of the detected ROAs [depicted as solid areas in panel (ii)]. **(C)** Signal and **(D)** ROA density. **(E)** Relative frequency of ROAs active once (lower bars) or more than once (upper bars) over the recording time. **(F)** ROA area and **(G)** mean relative number of simultaneously active ROAs over the total recording time calculated as coincidence index (*c.i.*). *Ex vivo* (*d0*) *wm* data were placed next to the *in vivo* data for direct comparison. **(C–G)** Data were represented as mean ± SEM of single FOVs. Single datasets were analyzed using a Shapiro–Wilk normality test and represented as FOV medians. Data were compared using an Ordinary one-way ANOVA with Bonferroni’s multiple comparisons test. Comparisons between *in vivo* and *ex vivo* data were displayed on top of the single bars. For each group in panel **(E)**, the relative numbers of ROAs presenting one or more signal peaks were analyzed using a Wilcoxon matched pairs signed rank test. *N* (animals) = 4 (astroglia, *d0*), 4-6-4 (astroglia, *in vivo*, *d1-d7*), 6 (microglia, *d0*), 4 (microglia, *in vivo*, *d1-d7*). *n* (FOVs) = 14 (astroglia, *d0*), 13-20-13 (astroglia, *in vivo*, *d1-d7*), 22 (microglia, *d0*), 14-13-10 (microglia, *in vivo*, *d1-d7*). *, ^#^, ^$^*p* < 0.05; ^**^, ^##^, ^$$^*p* < 0.01; ^***^, ^###^*p* < 0.001; ^***^p < 0.0001.

To provide a comprehensive study of astroglial and microglial Ca^2+^ events *in vivo* we used chronic 2P-LSM of the dorsal spinal cord *wm* tracts and compared them with the Ca^2+^ dynamics recorded in acute *wm* slice preparations (*ex vivo*). Following chronic spinal cord window implantation, hGFAP^GCaMP3^ and CX_3_CR_1_^GCaMP3^ mice were analyzed in slightly anesthetized conditions (1.5% isoflurane, 66% O_2_ and 33% N_2_O) at three different time points (*d1*, *d2*, and *d7*, Figure 4 and Supplementary Figure 1A). The cytosolic GCaMP3 expression confirmed the absence of any obvious structural reactive phenotype as previously shown by immunohistochemistry (Figures 4, 5A and Supplementary Videos 5–10). The quantification of spontaneous Ca^2+^ events revealed higher signal densities (10^–3^/μm^2^) for *wm* microglia *in vivo* (*d1*: *p* < 0.001; *d2*: *p* < 0.01; *d7*: *p* < 0.01) compared to acute slice preparations (*d0*). In contrast to that, astrocytes showed lower signal numbers *in vivo* at *d2* (*p* < 0.01) and *d7* (*p* < 0.05) compared to *ex vivo* preparations (*d0*), and for each time point compared to microglia *in vivo* (*p* < 0.01) (Figures 5A–C). In line with this, we found that astrocytes displayed a ∼2-fold reduction in ROA density (10^–3^/μm^2^) *in vivo* (*d1*: *p* < 0.05; *d2*: *p* < 0.0001; *d7*: *p* < 0.0001) compared to *ex vivo*. Notably, the astroglial ROA density decreased over time from acute (*d1*) to the chronic phase (*d7*) *in vivo*. Contrarily, *in vivo* microglia (*d1*: *p* < 0.001; *d2*: *p* < 0.01; *d7*: *p* < 0.05) displayed a twofold increase in ROA density compared to *ex vivo* recordings as well as to astrocytes from *d2* (*d2*: *p* < 0.0001; *d7*: *p* < 0.0001). This finding showed an opposite trend from the *ex vivo* slice preparations, where we found lower ROA density for microglia compared to astroglia (Figure 5D). When we looked at the relative number of ROAs (%) associated with either one or more peaks during the acquisition, we found, in contrast to their equal distribution in *ex vivo* recordings, an almost twofold higher percentage of ROAs with only one peak compared to the ROAs with more than one peak for astrocyte recordings *in vivo*. On the other hand, microglial Ca^2+^ signals displayed an opposite phenotype with a higher percentage of ROAs characterized by more than one peak (Figure 5E). The signal frequency (min^–1^) of the active ROAs (>1 signal peak) did not display any difference for *in vivo* or *ex vivo* recordings or between the cell-types (Table 2).

No difference was detected in terms of signal amplitude as well as signal duration (Table 2). In line with this, the relative frequency distribution of glial signal amplitudes displayed only minor or no differences for astroglia (*d1*∼*d2*∼*d7*) and compared to microglia. In contrast to astrocytes, the signal amplitudes of microglia varied among chronic recordings (*p* < 0.0001) and compared to *ex vivo* slice recordings (*d1*: *p* < 0.001; *d2*: *p* < 0.0001; *d7*: *p* < 0.0001). Notably, the difference of microglial amplitude frequency distributions between *in vivo* and *ex vivo* conditions increased over time from *d1* to *d7* (Supplementary Figure 3A). The signal durations of astrocytes were longer *in vivo* than *ex vivo* but became more similar along the investigated time points (*d1*: *p* < 0.0001; *d2*: *p* < 0.0001; *d7*: *p* < 0.0001). Furthermore, microglia displayed the longest signal durations at *d1* in comparison to *d2* (*p* < 0.0001) and *d7* (*p* < 0.001) but also to acute *ex vivo* slice preparations (*p* < 0.0001) and regarding to astroglia (*d1*, *p* < 0.0001, Supplementary Figure 3B). As it was the case for the acute slice preparations, the integrated fluorescence of the Ca^2+^ signals as well as their rise and decay time did not display any difference either between *in vivo* and *ex vivo* recordings or between the two cell types (Table 2). The area (μm^2^) of *in vivo* Ca^2+^ signals was more than five times larger than *ex vivo* for astrocytes (*d1*: *p* < 0.01; *d2*: *p* < 0.05; *d7*: *p* < 0.0001) and at least four times larger for microglia. In particular, we found that both astroglia and microglia displayed a larger ROA area 1 week after the window implantation (*d7*), whereas in the acute phase (*d1-d2*) only astroglial signals were associated with larger ROA areas (Figure 5F). The relative frequency distribution of the ROA areas also showed that *in vivo* astrocytes (*p* < 0.0001) and microglia (*p* < 0.0001) were highly different to *ex vivo* recordings. On the other hand, there were only minor differences among the astroglial datasets *in vivo* and between the early *in vivo* recordings of microglia (*d1-d2*). We found no difference between astroglia and microglia at *d7*, but the ROA area was strongly reduced in microglia compared to astrocytes at *d1* and *d2* (*p* < 0.0001, Supplementary Figure 3C). We then assessed the relative number of simultaneously active ROAs in the FOV space, revealing two to three times more coincidently active ROAs in *in vivo* recordings in microglia compared to astroglia (Figure 5G).

To finalize our comparison, we performed a cluster analysis on all parameters provided by MSparkles, aiming at identifying specific segregation patterns within the presented data (Supplementary Figure 4). Since we could not clearly distinguish microglia and astrocytes (Supplementary Figure 4A), we separated the two cell types and could find only a partial segregation of the microglial data between *ex* and *in vivo* recordings (Supplementary Figures 4B,C).

## Discussion

In this work, we determined the characteristics of physiological Ca^2+^ dynamics of astrocytes and microglia in the murine spinal cord employing *in vivo* and *ex vivo* two-photon laser-scanning microscopy (2P-LSM) of transgenic mice expressing the genetically encoded Ca^2+^ indicator GCaMP3. To compare glial Ca^2+^ dynamics, we took advantage of a custom-made MATLAB-based analysis software (MSparkles) that identifies fluorescence changes in an unbiased and morphology independent manner and determines signal peak as well as region of activity (ROA) associated parameters. Our analysis revealed that microglia have a strongly reduced signal as well as ROA density in *ex vivo* preparations compared to astroglia (Figures 3C,D). Notably, this is not associated with overall differences in astro- and microglial signal frequency within each active ROA (Figure 3E and Table 2). In contrast to this, *in vivo* microglia show higher signal and ROA density than astrocytes (Figures 5C,D). This opposite findings between *ex vivo* and *in vivo* recordings may be due to an activation of astrocytes and microglia resulting from the excessive manipulation required for spinal cord extraction and acute slice preparation resulting in an excessively high (astrocytes) or excessively low (microglia) Ca^2+^ activity in line with the change of microglia morphology to a more amoeboid appearance in slices. It was recently shown for the brain that astroglial Ca^2+^ dynamics differ between *ex vivo* and *in vivo* (as well as *in situ*) recordings (Müller et al., 2021) supporting our results. Astroglial ROA densities decrease *in vivo* along the imaging sessions, possibly hinting that higher ROA densities are associated with an alteration of astroglial signaling in the acute phase after laminectomy non-detectable by reactive markers. Also, glial Ca^2+^ signaling is differentially affected by anesthesia used during the *in vivo* recording. In line with our observations, previous work shows a reduced Ca^2+^ activity in astroglia both in brain and spinal cord (Thrane et al., 2012; Poskanzer and Yuste, 2016; Sekiguchi et al., 2016; Schweigmann et al., 2021) and an increased Ca^2+^ activity in microglia in the brain under anesthesia (Umpierre et al., 2020). Although microglial ROA density is higher *in vivo*, the ROA area itself is smaller than for astrocytes both *ex vivo* and *in vivo* in the early phase (*d1* and *d2*) after the window implantation (Figures 3F, 5F), suggesting that activated microglial Ca^2+^ dynamics are reduced compared to physiological conditions. In line with this, our recordings showed that astroglia display concerted Ca^2+^ waves beyond the extension of single cells, whereas microglial Ca^2+^ signaling is mainly restricted to single processes in acute slices and directly after spinal cord window surgery.

In terms of 3D structural properties of the single Ca^2+^ changes (amplitude, duration, integrated fluorescence, rise and decay time), we found no substantial differences between astrocytes and microglia as well as between *ex vivo* and *in vivo* recordings (Table 2). We conclude that the characterization of spontaneous Ca^2+^ changes in terms of commonly assessed parameters (as amplitude and duration) or even more sophisticated geometrical descriptive parameters (such as integrated fluorescence, rise and decay time) cannot enable a successful segregation of glial Ca^2+^ dynamics even if collectively assessed as we did using a clustering analysis (Supplementary Figure 4). This suggests that glial cells may share some common mechanisms underlying Ca^2+^ signaling or that cell-specific pathways originate similar cytosolic Ca^2+^ elevations and that the different glial Ca^2+^ dynamics arise from the temporal and spatial control of otherwise similar signals at the cellular or network level. Indeed, it is known since decades that glial cells share some common mechanisms at the basis of intracellular Ca^2+^ mobilization (Verkhratsky and Kettenmann, 1996; Deitmer et al., 1998). Nevertheless, pharmacological as well as genetic approaches aiming at interfering with putative molecular mechanisms underlying Ca^2+^ fluctuations are required to draw any conclusion on this point.

In acute spinal cord preparations, microglia display similar Ca^2+^ signaling properties between gray (*gm*) and white matter (*wm*; Figure 3) whereas astroglial Ca^2+^ signals last longer (Table 2) and have a larger extension (Figure 3F) in *gm* compared to *wm*. This finding suggests that diverse Ca^2+^ dynamics might be due to the morphological heterogeneity of astroglia that can be clearly observed between *gm* and *wm* (Figure 1) in line with previous evidence obtained in the brain showing less coupling of *wm* fibrous astrocytes (mainly though connexin 43) compared to *gm* protoplasmic astroglia (through connexins 43 and 30) (Lee et al., 1994; Haas et al., 2006). In addition, astroglial Ca^2+^ waves in *wm*, in contrast to the neocortical *gm*, mainly propagate through ATP release (Schipke et al., 2002; Hamilton et al., 2008). Also, *gm* and *wm* astroglia receive different glutamatergic inputs given their close proximity with the neuronal synapses in the *gm* and the relatively lower level of glutamate release from neuronal axons in the *wm* (Kukley et al., 2007; Ziskin et al., 2007; Wake et al., 2011). Taken together, these data point at the existence of a regional specificity for astroglial Ca^2+^ signaling which reflects the cellular heterogeneity between *gm* and *wm* (Köhler et al., 2021). On the other hand, microglial Ca^2+^ dynamics display a substantial uniformity between *gm* and *wm*, in line with the absence of morphological regional differences.

The monitoring of astroglial and microglial Ca^2+^ dynamics for up to 7 days after laminectomy and chronic window implantation enabled the evaluation of putative specific glial responses to the manipulation required for *in vivo* imaging (Figure 5). In particular, microglia display a more differential phenotype between the acute phase (*d1* and *d2*) after laminectomy and the later chronic phase (*d7*) than astroglia. Microglia signals are characterized by higher duration and smaller area in the acute phase and with higher amplitude, lower duration and larger area in the chronic phase. Moreover, a closer look at the relative signal frequency distributions revealed higher similarities for astroglia in terms of amplitude as well as duration, whereas microglial signal distribution displayed a high heterogeneity along the recording time points. Notably, signal amplitude and duration distribution of astroglia and microglia become more and more similar with time. Similar findings were obtained from the analysis of the distribution of the ROA areas, thus suggesting that *in vivo* glial cell are similar in the chronic phase but differ after acute activation following the perturbation of the surrounding environment. This is in line with evidence supporting the different response kinetics of spinal cord microglia (Prewitt et al., 1997; Dibaj et al., 2010; Bellver-Landete et al., 2019) and astrocytes (Okada et al., 2006; Fan et al., 2016; Li et al., 2019, 2020) in response to external stimuli. This may also underline the differences observed between astroglia and microglia in acute slice preparations, since the experimental procedure required to collect the tissue constitutes a significant challenge to the physiology of the spinal cord. In line with this, microglial signal amplitude distribution becomes progressively more different than the signal distribution from *ex vivo* recordings along the experimental time points, suggesting a progressive restoration of the physiological status disrupted after spinal cord slice collection or window implantation. To test the nature of glial Ca^2+^ reactive phenotype, we propose to acutely challenge glial Ca^2+^ dynamics *in vivo* by means of focal application of mechanical (e.g., laser induced) as well as chemical (e.g., DAMPs such as ATP) perturbative stimuli to trigger event-based Ca^2+^ signaling.

Finally, with respect to the temporal dynamics of astro- and microglial Ca^2+^ signaling, we showed that both glial cell populations are associated with the same signal frequency but different activity (i.e., relative number of ROAs active once or more than once during the recording time) (Figure 5E) as well as a different extent of coincident activity (Figure 5G). In particular, the relative frequency of astroglial ROAs with more than one peak *in vivo* is higher compared to microglial ROAs, while the signal frequency is not changed between the two cell types. In parallel to that, astroglia display a lower coincidence index compared to microglia. The enhanced microglial coincidence *in vivo* is likely due to their constant dynamic scanning of the surrounding environment since microglial Ca^2+^ changes were shown to precede cell motility in the brain (Umpierre et al., 2020). In addition to that, glial Ca^2+^ dynamics might be the result of a differential cell responsivity to their neuronal counterpart. This study was conceptualized minimizing the interference of the neuronal network *in vivo* by means of isoflurane anesthesia, thus avoiding the superimposition of a further level of complexity arising from motor as well as sensory stimuli. Nevertheless, the evaluation of glial Ca^2+^ dynamics in awake animals is required to fully elucidate glial heterogeneity and we strongly believe that the evaluation of event triggered Ca^2+^ signaling (like in response to the induction of reflex circuits) will enable the identification of a further layer of glial specificity in terms of Ca^2+^ dynamics.

## Conclusion

In summary, we performed two-photon laser-scanning microscopy in acute slice preparations and chronic *in vivo* recordings of the mouse spinal cord to simultaneously characterize and compare cell-specific properties of Ca^2+^ signals in astro- and microglia. To this aim, we used specific user-defined parameters and a novel analysis tool to evaluate common and distinct features of spinal glial cells with respect to their physiological Ca^2+^ changes. Accordingly, we conclude that signal and ROA density, ROA area, signal frequency and coincidence are key parameters for the differentiation of glial Ca^2+^ dynamics and are therefore valuable candidates for understanding the highly developed function of astrocytes and microglia in the environment of the mammalian spinal cord. Acute slice preparation as well as the spinal cord surgery influence both astro- and microglial Ca^2+^ dynamics and we found that microglia, as predictable given their surveillance activity, are more susceptible to the experimental manipulation. Nevertheless, the reliable and standardized analysis of Ca^2+^ dynamics remains an open challenge to be addressed in the near future to fully elucidate the role of spinal glial cells in physiology and pathology.

## Data Availability Statement

The raw data supporting the conclusions of this article will be made available by the authors, without undue reservation.

## Ethics Statement

The animal study was reviewed and approved by Saarländisches Landesamt für Gesundheit und Verbraucherschutz.

## Author Contributions

PR and DG equally contributed to the manuscript, conceptualized the project, performed the experiments, analyzed the data, wrote the first draft, and generated the figures. PR performed laminectomy and spinal cord window implantation and prepared the spinal cord tissue for further processing. DG obtained and handled the spinal cord acute preparations. GS developed the ROA-based automatic analysis for image processing. AW contributed to the data analysis and visualization. ED contributed to the data acquisition. FK provided the structural and financial support for the project. AS conceptualized and supervised the project, reviewed and finalized the manuscript and figures. All authors approved on the final version of the manuscript.

## Conflict of Interest

The authors declare that the research was conducted in the absence of any commercial or financial relationships that could be construed as a potential conflict of interest.

## Publisher’s Note

All claims expressed in this article are solely those of the authors and do not necessarily represent those of their affiliated organizations, or those of the publisher, the editors and the reviewers. Any product that may be evaluated in this article, or claim that may be made by its manufacturer, is not guaranteed or endorsed by the publisher.

## Footnotes

1
http://www.rstudio.com/

2
https://CRAN.R-project.org/package=pheatmap

## Supplementary Material

Supplementary Figure 1 Chronic spinal cord window implantation induced no abnormal glial cell reactivity. **(A)** Experimental design for the evaluation of the chronic glial cell reactivity after laminectomy and spinal cord window implantation with 10-weeks old mice injected with tamoxifen (TAM) and processed 2 weeks later for either immunohistochemistry (IHC, *d0*) or laminectomy surgery for chronic spinal cord window implantation and subsequently perfusion after 1, 2, or 7 days (*d1*, *d2*, and *d7*). **(B)** Bright field overview of spinal cord tissue exposed through laminectomy and monitored for up to 7 days and relative magnification of the selected areas (i–iii). The regions in correspondence of the removed spinal vertebrae (T13 and L1, left) and the underlying spinal cord segments (L4-L6, S1; right) are indicated. Scale bar, 1 mm (overviews) and 200 μm (magnified views). **(C)** Evaluation of astroglial and microglial reactivity in mice expressing GCaMP3 in astrocytes (blue) or microglia (yellow) stained for GFAP (glial fibrillary acidic protein, red) and Iba1 (green) for astroglial and microglial reactivity, respectively. Cell nuclei were stained with DAPI (blue). Scale bar, 200 μm.

Supplementary Figure 2 *Ex vivo* distribution of glial signal amplitudes, durations and ROA areas. **(A)** Relative frequency of signal amplitude (divided into 0.1 ΔF/F_0_ bins), **(B)** signal duration, divided 2 s bins) and **(C)** ROA area (divided into 5 μm^2^ bins) for astroglia (blue) and microglia (yellow) in *gm* (solid) and *wm* (dashed) of acute slice preparations. Data with peak amplitudes greater than 0.7 ΔF/F_0_ (0.7 ± 0.5%), durations longer than 40 s (0.1 ± 0.1%) and ROA area greater than 120 μm^2^ (0.6 ± 0.2%) were excluded from the representation for easier display. Data were represented as mean ± SEM, fitted with a lognormal curve using a Least-Squares fitting with no weighting method and compared using the extra-sum-of-squares F test. F ratios and relative *p-values* of single curve comparisons were schematically represented as a squared diagram and gray-scaled color-coded. Smaller insets represent magnified views of the fitting curves. *N* (animals) = 4 (astroglia), 5 (microglia, *gm*), 6 (microglia, *wm*). *n* (FOVs) = 12 (astroglia, *gm*), 14 (astroglia, *wm*), 15 (microglia, *gm*), 22 (microglia, *wm*). ^****^*p* < 0.0001.

Supplementary Figure 3 *In vivo* distribution of glial signal amplitudes, durations and ROA areas. **(A)** Relative frequency of signal amplitude (divided into 0.15 ΔF/F_0_ bins), **(B)** signal duration (divided into 2 s bins) and **(C)** ROA area (divided into 25 μm^2^ bins) for astroglia (blue) and microglia (yellow) *in vivo* (*d1*, *d2* and *d7*, *wm*) and *ex vivo* (*d0*, *wm*). Data with peak amplitude greater than 0.5 ΔF/F_0_ (3.3 ± 1.3%), duration longer than 40 s (0.4 ± 0.3%) and ROA areas larger than 400 μm^2^ (5.3 ± 1.8%) were excluded from the representation for easier display. Data were represented as mean ± SEM, fitted with a lognormal curve using a Least-Squares fitting with no weighting method and compared using the extra-sum-of-squares F test. F ratios and relative *p-values* of single curve comparisons were schematically represented as a triangle diagram and gray-scaled color-coded. Non-significantly different curves were plotted as a shared curve (*d1*∼*d2*∼*d7*). Smaller insets represent magnified views of the fitting curves. *N* (animals) = 4 (astroglia, *d0*), 4-6-4 (astroglia, *in vivo*, *d1-d7*), 6 (microglia, *d0*), 4 (microglia, *in vivo*, *d1-d7*). *n* (FOVs) = 14 (astroglia, *d0*), 13-20-13 (astroglia, *in vivo*, *d1-d7*), 22 (microglia, *d0*), 14-13-10 (microglia, *in vivo*, *d1-d7*). **p* < 0.05; ^**^*p* < 0.01; ^***^*p* < 0.001; ^****^*p* < 0.0001.

Supplementary Figure 4 Hierarchical clustering of Ca^2+^ signalling data. **(A)** Heatmap of the unsupervised hierarchical clustering of Ca^2+^ signaling data represented as medians of single FOVs and color-coded based on the row *z*-scores for both cell-types, **(B)** astroglia or **(C)** microglia. Datasets were color coded based on cell-type (astroglia, blue; microglia, yellow), experimental procedure (*ex vivo*, light blue; *in vivo*, orange) or spinal cord region (*gm*, gray; *wm*, white).
