# Supplementary figures and images for: Astrocytes and Microglia Exhibit Cell-Specific Ca2+ Signaling Dynamics in the Murine Spinal Cord
Source: Front Mol Neurosci. 2022 Mar 30;15:840948. doi: 10.3389/fnmol.2022.840948 (PMC9006623; doi:10.3389/fnmol.2022.840948)

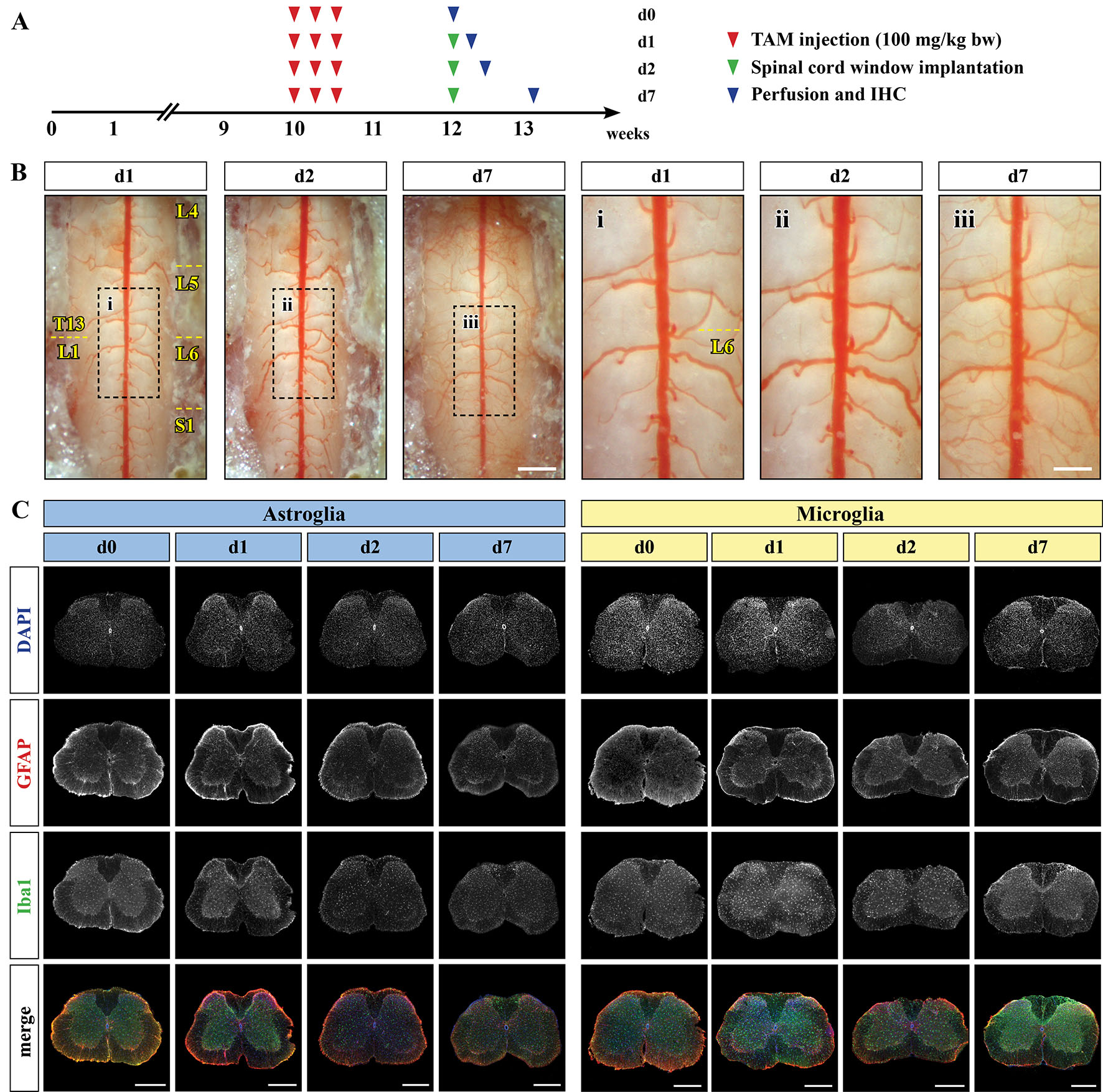

Supplement: Supplementary Figure 1 — Chronic spinal cord window implantation induced no abnormal glial cell reactivity. (A) Experimental design for the evaluation of the chronic glial cell reactivity after laminectomy and spinal cord window implantation with 10-weeks old mice injected with tamoxifen (TAM) and processed 2 weeks later for either immunohistochemistry (IHC, d0) or laminectomy surgery for chronic spinal cord window implantation and subsequently perfusion after 1, 2, or 7 days (d1, d2, and d7). (B) Bright field overview of spinal cord tissue exposed through laminectomy and monitored for up to 7 days and relative magnification of the selected areas (i–iii). The regions in correspondence of the removed spinal vertebrae (T13 and L1, left) and the underlying spinal cord segments (L4-L6, S1; right) are indicated. Scale bar, 1 mm (overviews) and 200 μm (magnified views). (C) Evaluation of astroglial and microglial reactivity in mice expressing GCaMP3 in astrocytes (blue) or microglia (yellow) stained for GFAP (glial fibrillary acidic protein, red) and Iba1 (green) for astroglial and microglial reactivity, respectively. Cell nuclei were stained with DAPI (blue). Scale bar, 200 μm. [file Image_1.JPEG]

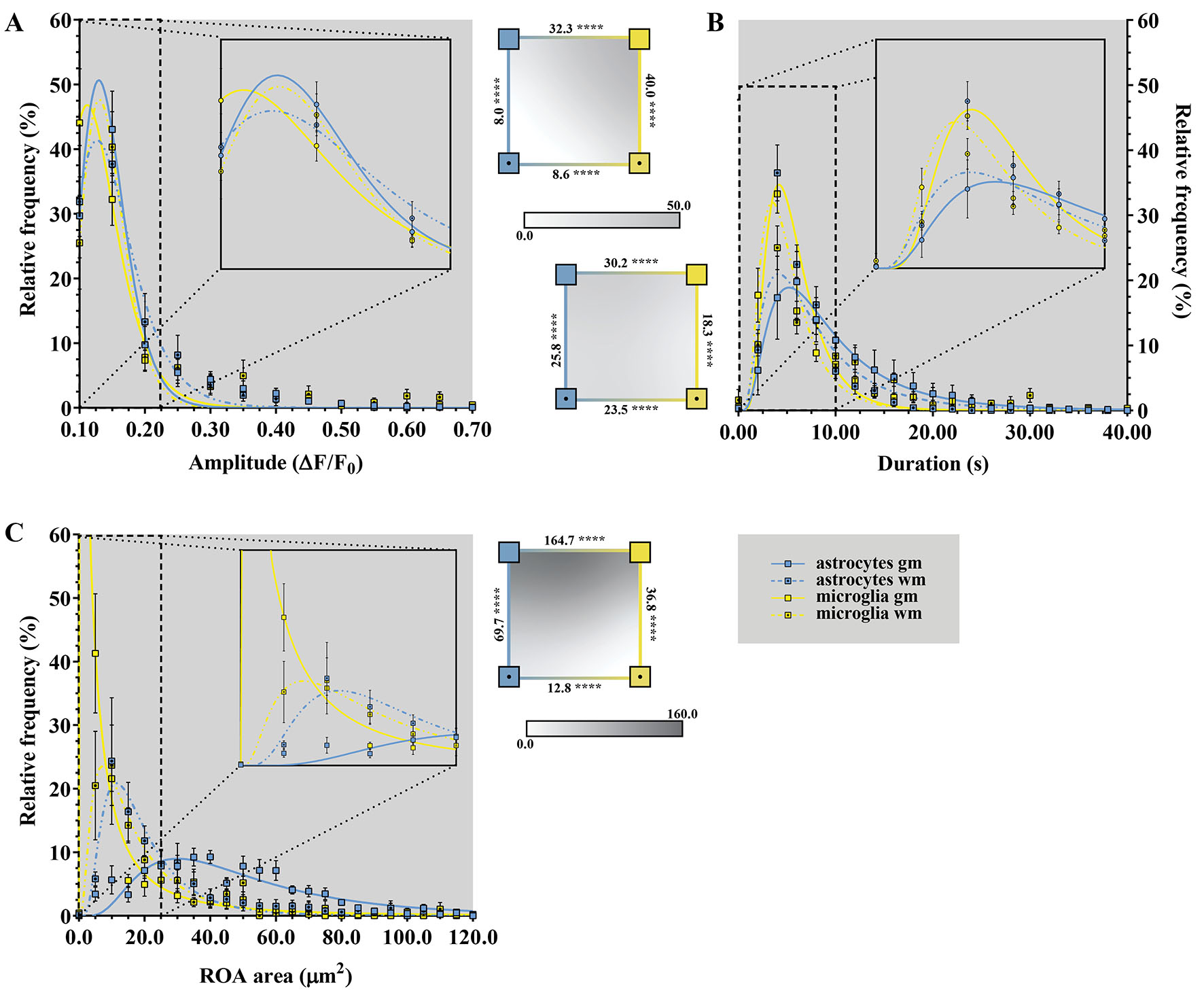

Supplement: Supplementary Figure 2 — Ex vivo distribution of glial signal amplitudes, durations and ROA areas. (A) Relative frequency of signal amplitude (divided into 0.1 ΔF/F0 bins), (B) signal duration, divided 2 s bins) and (C) ROA area (divided into 5 μm2 bins) for astroglia (blue) and microglia (yellow) in gm (solid) and wm (dashed) of acute slice preparations. Data with peak amplitudes greater than 0.7 ΔF/F0 (0.7 ± 0.5%), durations longer than 40 s (0.1 ± 0.1%) and ROA area greater than 120 μm2 (0.6 ± 0.2%) were excluded from the representation for easier display. Data were represented as mean ± SEM, fitted with a lognormal curve using a Least-Squares fitting with no weighting method and compared using the extra-sum-of-squares F test. F ratios and relative p-values of single curve comparisons were schematically represented as a squared diagram and gray-scaled color-coded. Smaller insets represent magnified views of the fitting curves. N (animals) = 4 (astroglia), 5 (microglia, gm), 6 (microglia, wm). n (FOVs) = 12 (astroglia, gm), 14 (astroglia, wm), 15 (microglia, gm), 22 (microglia, wm). ****p < 0.0001. [file Image_2.JPEG]

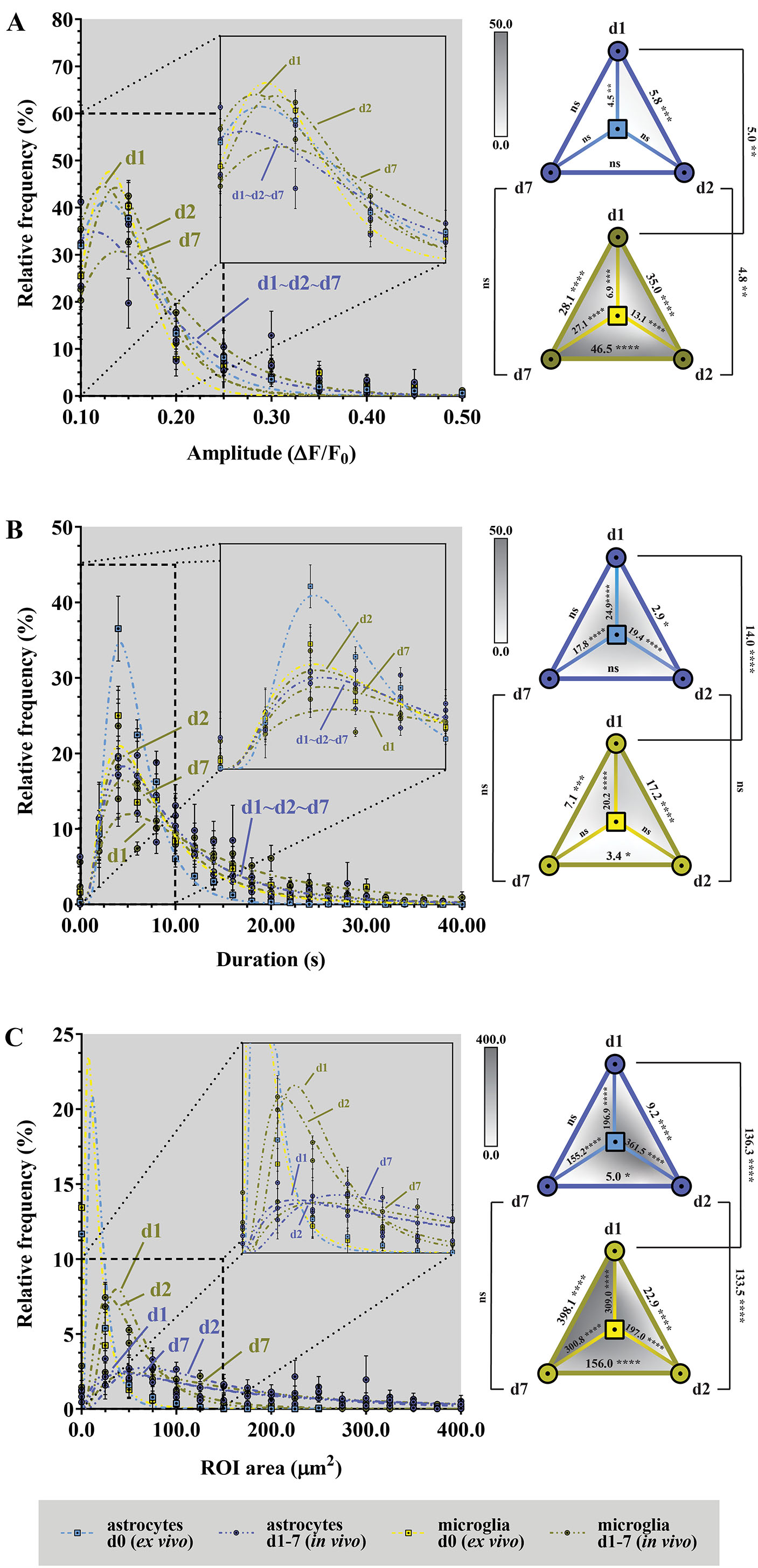

Supplement: Supplementary Figure 3 — In vivo distribution of glial signal amplitudes, durations and ROA areas. (A) Relative frequency of signal amplitude (divided into 0.15 ΔF/F0 bins), (B) signal duration (divided into 2 s bins) and (C) ROA area (divided into 25 μm2 bins) for astroglia (blue) and microglia (yellow) in vivo (d1, d2 and d7, wm) and ex vivo (d0, wm). Data with peak amplitude greater than 0.5 ΔF/F0 (3.3 ± 1.3%), duration longer than 40 s (0.4 ± 0.3%) and ROA areas larger than 400 μm2 (5.3 ± 1.8%) were excluded from the representation for easier display. Data were represented as mean ± SEM, fitted with a lognormal curve using a Least-Squares fitting with no weighting method and compared using the extra-sum-of-squares F test. F ratios and relative p-values of single curve comparisons were schematically represented as a triangle diagram and gray-scaled color-coded. Non-significantly different curves were plotted as a shared curve (d1∼d2∼d7). Smaller insets represent magnified views of the fitting curves. N (animals) = 4 (astroglia, d0), 4-6-4 (astroglia, in vivo, d1-d7), 6 (microglia, d0), 4 (microglia, in vivo, d1-d7). n (FOVs) = 14 (astroglia, d0), 13-20-13 (astroglia, in vivo, d1-d7), 22 (microglia, d0), 14-13-10 (microglia, in vivo, d1-d7). *p < 0.05; **p < 0.01; ***p < 0.001; ****p < 0.0001. [file Image_3.JPEG]

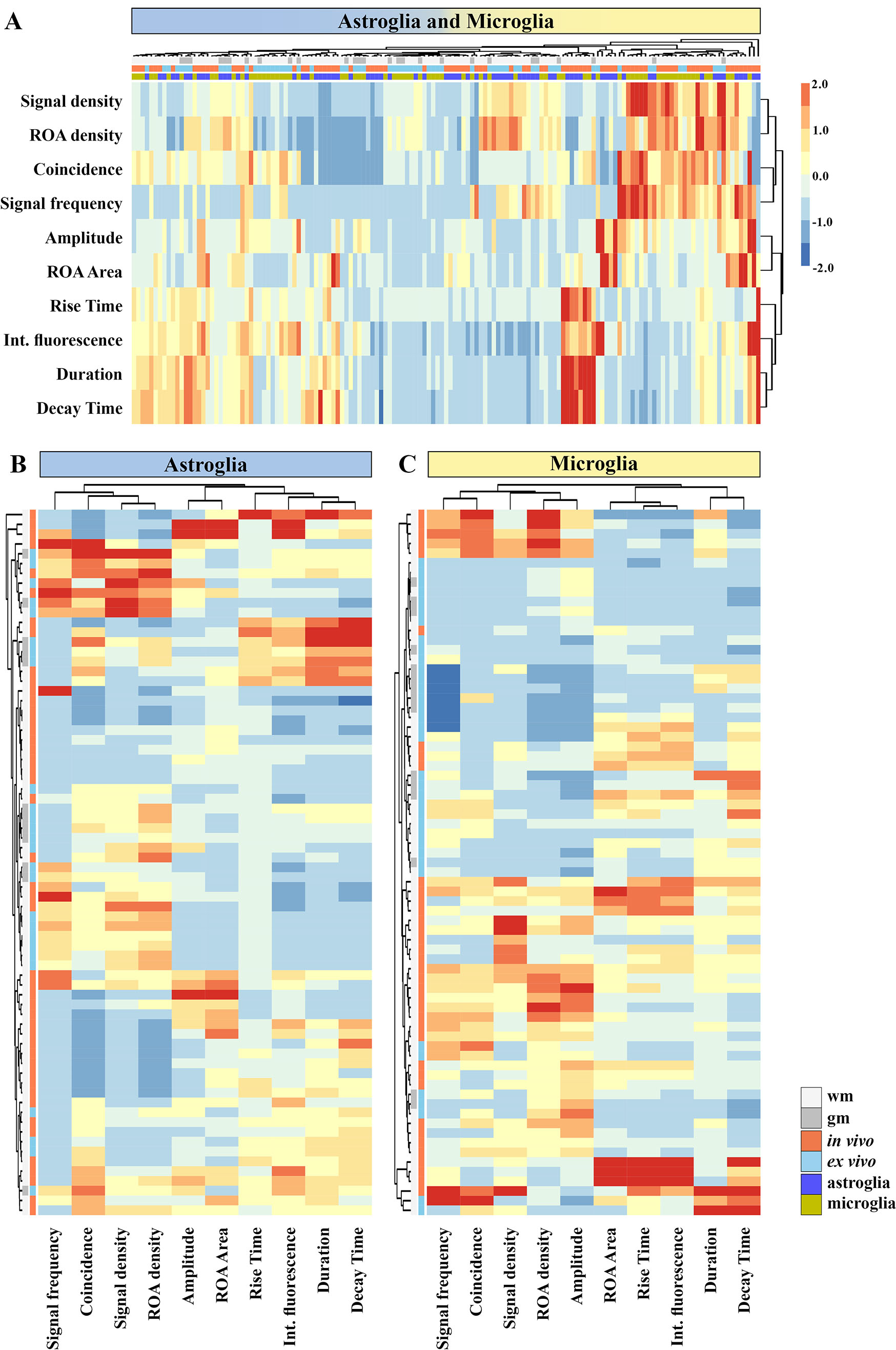

Supplement: Supplementary Figure 4 — Hierarchical clustering of Ca2+ signalling data. (A) Heatmap of the unsupervised hierarchical clustering of Ca2+ signaling data represented as medians of single FOVs and color-coded based on the row z-scores for both cell-types, (B) astroglia or (C) microglia. Datasets were color coded based on cell-type (astroglia, blue; microglia, yellow), experimental procedure (ex vivo, light blue; in vivo, orange) or spinal cord region (gm, gray; wm, white). [file Image_4.JPEG]
